# Direct, minimally intrusive quantification of rebreathing in the circle breathing system during spontaneous breathing: a proof-of-concept feasibility study

**DOI:** 10.3389/fmed.2026.1780197

**Published:** 2026-07-08

**Authors:** Jie Cui, Yanling Tan, Pan Li

**Affiliations:** Department of Anesthesiology, Children’s Hospital of Chongqing Medical University, National Clinical Research Center for Children and Adolescents’ Health and Diseases, Ministry of Education Key Laboratory of Child Development and Disorders, Chongqing Key Laboratory of Child Neurodevelopment and Cognitive Disorders, Chongqing, China

**Keywords:** breathing tubing, circle breathing system, ideal gas, rebreathing, spontaneous breathing

## Abstract

**Background:**

During spontaneous breathing in the circle breathing system, hypercapnia can occur partly due to the rebreathing in the breathing tubing; however, there is currently no explicit method to quantify this specific portion of rebreathing.

**Methods:**

We derived formulas from the ideal gas law to calculate the rebreathing. To conduct an initial proof-of-concept feasibility assessment, we applied these formulas to data from only one pediatric patient—a 5-year-old child under general anesthesia. Spontaneous breathing was maintained with fresh gas flow of 2, 4, and 6 L/min, with real-time monitoring of gas states within the breathing tubing.

**Results:**

The total maximum rebreathing volumes originating from the breathing tubing were 0.54 ± 0.05 mL, 0.47 ± 0.03 mL, and 0.43 ± 0.03 mL, respectively, showing a decreasing trend with increasing fresh gas flow.

**Conclusion:**

Spontaneous breathing via the circle breathing system leads to rebreathing from the breathing tubing, and the specific value of the rebreathing resulting from the breathing tubing can be calculated using the formulas obtained from the ideal gas law.

## 1 Introduction

The circle breathing system is currently the most used method, especially in inhalation sedation, due to its outstanding comprehensive advantages (1). Its reliability in mechanical ventilation has been fully recognized, making it applicable to general anesthesia for almost all patients, including neonates (2). However, the third edition of *Anesthesia Equipment: Principles and Applications* notes that when the circle breathing system switches from mechanical ventilation to spontaneous breathing, it causes an additional increase in rebreathing, with part of the breathing tubing becoming apparatus dead space. This is one of the significant concerns regarding the safe application of the circle breathing system in patients maintaining spontaneous breathing, especially in pediatric patients (3).

Hypercapnia will occur if rebreathing due to dead space in ventilation is beyond the body’s compensatory ability (4). It is known that children who breathed spontaneously via the circle breathing system were more likely to develop hypercapnia (5, 6). This hypercapnia occurred even when the minute ventilation was adequate to meet both the average oxygen consumption of the children (7) and the estimated dead space within the breathing system (8), as well as when the established fresh gas flow was theoretically sufficient (9). However, such spontaneous breathing can not only avoid the risks associated with endotracheal intubation and extubation but also meet the requirements of the tubeless technique (10, 11) and the enhanced recovery after surgery (12), as well as fully leverage the advantages of inhalation sedation (13, 14). Therefore, minimizing the rebreathing originating from the breathing tubing may hold clinical importance for improving the safety of spontaneous breathing via the circle breathing system.

In our previous work, we modified the breathing tubing and validated through piglet experiments that this design somewhat reduced carbon dioxide accumulation in the body during spontaneous breathing via the circle breathing system (15). However, due to the lack of effective methods for monitoring changes in fluid states within the breathing tubing—specifically, monitoring approaches that do not interfere with the flowing gas in the main pathways—the study could not clarify whether the improved efficiency of carbon dioxide clearance was due to minimizing rebreathing from the tubing, nor could it further evaluate the implications for spontaneous breathing via the circle breathing system. We want this study to develop a novel method for calculating the rebreathing that originates from the breathing tubing.

## 2 Materials and methods

### 2.1 Ideal gas and formulas

**Figure 1:**
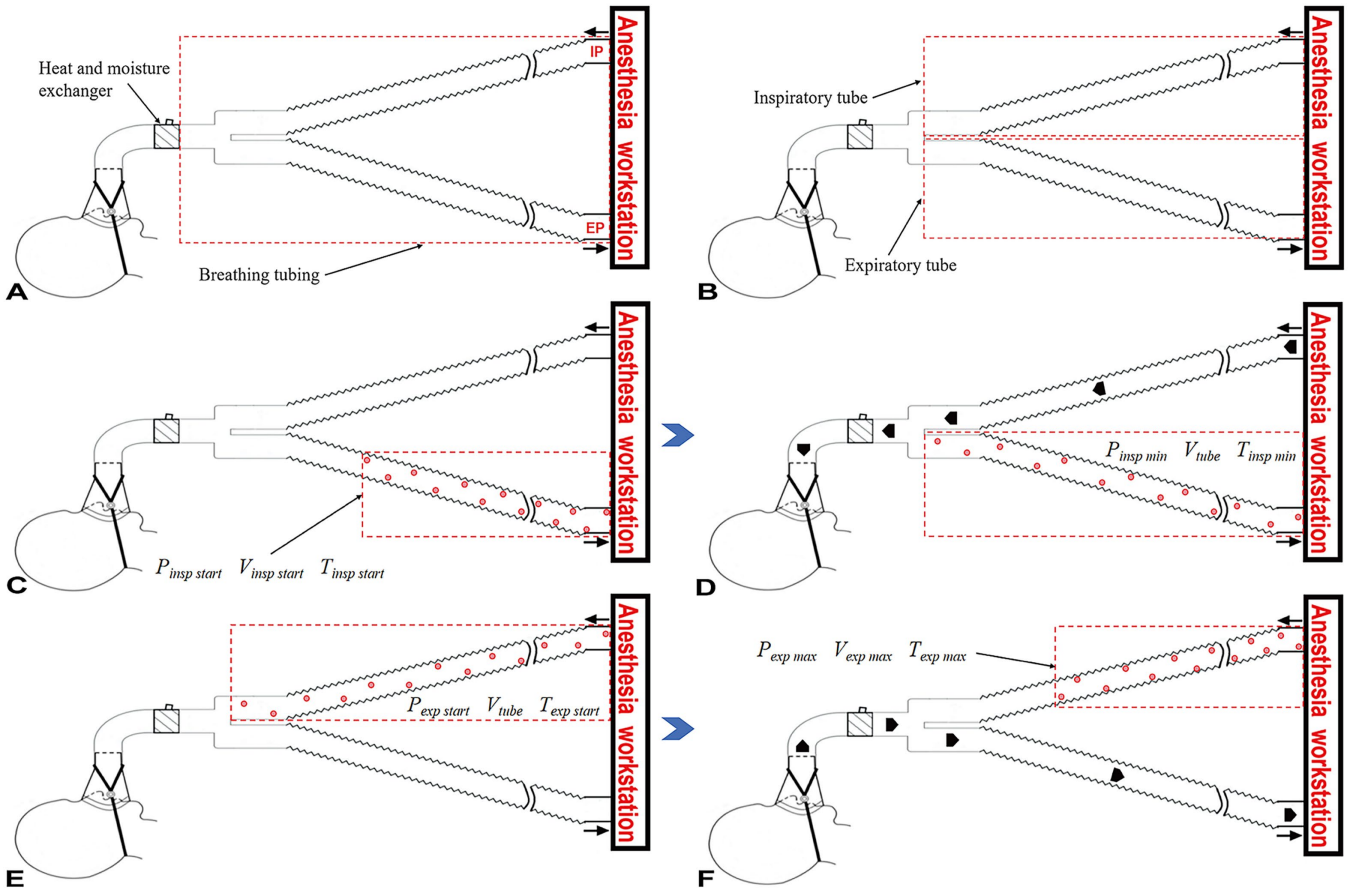
Ideal gas states change during spontaneous breathing via the breathing tubing. **(A)** A patient connected to the circle breathing system via the breathing tubing; **(B)** Inspiratory tube and expiratory tube; **(C)** Start of inspiration; **(D)** Moment of minimum pressure in the expiratory tube during inspiration; **(E)** Start of expiration; **(F)** Moment of maximum pressure in the inspiratory tube during expiration. “←” indicates the direction of gas flow at the inspiratory port of the anesthesia workstation; “→” indicates the direction of gas flow at the expiratory port of the anesthesia workstation; The thick black arrows inside the breathing tubing of d and f show the gas flow direction of spontaneous breathing; The small red circles indicate the ideal gas, and the red dashed boxes indicate the range of the ideal gas and the values of states. EP, expiratory port of the anesthesia workstation; IP, inspiratory port of the anesthesia workstation; *P_exp max_*, the maximum pressure in the inspiratory tube during expiration; *P_exp start_*, the pressure in the inspiratory tube at the start of expiration; *P_insp min_*, the minimum pressure in the expiratory tube during inspiration; *P_insp start_*, the pressure in the expiratory tube at the start of inspiration; *T_exp max_*, the temperature of the inspiratory tube at maximum pressure during expiration; *T_exp start_*, the temperature of the inspiratory tube at the start of expiration; *T_insp min_*, the temperature of the expiratory tube at minimum pressure during inspiration; *T_insp start_*, the temperature of the expiratory tube at the start of inspiration; *V_exp max_*, the volume of ideal gas in the inspiratory tube at maximum pressure during expiration; *V_insp start_*, the volume of ideal gas in the expiratory tube at the start of inspiration; *V_tube_*, the volume of the inspiratory/expiratory tube.

The anesthesia workstation, which is equipped with the circle breathing system, is connected to the breathing tubing via an inspiratory port and an expiratory port, each of which has a unidirectional valve (Figures 1A,B). These unidirectional valves ensure that gas flows only in the direction permitted by the valve openings, preventing reverse flow. According to this design, a portion of the gas within the breathing tubing is believed to experience only volume changes during the inspiration and expiration of spontaneous breathing. Our research focuses on these parts of the gas. During inspiration, the gas rebreathed by the patient is that which remains in the expiratory tube at the start of inspiration, after subtracting the gas under study that we mentioned above (Figures 1C,D). During expiration, some of the patient’s exhaled gas enters the inspiratory tube and may be subsequently rebreathed by the patient during the next inspiration (Figures 1E,F). This situation also implies that the gas rebreathed from the breathing tubing does not form a stable flow. Using direct measurement methods requires taking into account numerous complex and precise data points to be substituted into the fundamental equations of fluid dynamics (16), making it extremely difficult to implement in practice.

This gas, which we study, primarily consists of air, oxygen, carbon dioxide, and a small amount of water vapor. The temperature of this gas mixture varies within a narrow range close to the operating room temperature, and its pressure also varies within a narrow range near atmospheric pressure. It can be treated as an ideal gas with negligible error (17). The physical changes of this gas mixture comply with the ideal gas law.

The product of the pressure (*P*) and volume (*V*) of the ideal gas, divided by temperature (*T*), equals the product of the number of moles of gas (*n*) and the gas constant (*R*) (18) (Equation 1).


$$\frac{\mathrm{PV}}{T}=\mathrm{nR}$$ (1)

The *R* in the ideal gas law depends on the molar mass (kg/mol) of the gas mixture (18), meaning that variations in gas composition determine the value of *R*.

During inspiration (Figures 1C,D): Gas in the expiratory tube flows out of the system. The composition of this gas remains unchanged during the measurement period, so the gas constant *R* is constant.

During expiration (Figures 1E,F): First, the preliminary experiments show that the average increase in pressure within the inspiratory tube is only 0.1–0.11%. This pressure increase is positively correlated with the increase in the number of gas molecules, indicating that only a very small amount of exhaled gas enters the inspiratory tube during the study period, and its effect on *R* is minimal. Second, theoretically, the difference between the exhaled gas entering the inspiratory tube and the original gas in the inspiratory tube lies in water vapor, oxygen, and carbon dioxide. The use of a heat and moisture exchanger means that only a small amount of water vapor enters the inspiratory tube. Moreover, in pediatric patients, the oxygen consumed and carbon dioxide produced per breath constitute a very small fraction of the tidal volume of a single breath. Consequently, the difference in molar mass—and therefore in *R*—between the exhaled gas entering the inspiratory tube and the original gas in the inspiratory tube is very small.

In addition, the *n* we study remains constant. Meaning, the product of the *P* and *V* of the ideal gas, divided by *T*, is constant. Both pressure and temperature can be determined through measurements. The expiratory/inspiratory tube for children has an average diameter of 1.55 cm and a length of 1.2 m, resulting in a total volume of 226.3 mL.

At the start of inspiration, let the pressure, volume, and temperature of the ideal gas within the expiratory tube be denoted as *P_insp start_*, *V_insp start_*, and *T_insp start_*, respectively (Figure 1C). This ideal gas will fully occupy the entire expiratory tube when the pressure in it reaches its minimum during the inspiration. At this point, its volume equals the total volume of the expiratory tube (*V_tube_*), and its pressure and temperature are *P_insp min_* and *T_insp min_*, respectively (Figure 1D) (Equation 2).


$$\frac{P_{\text{insp start}}V_{\text{insp start}}}{T_{\text{insp start}}}=\frac{P_{\text{insp}\;\min}V_{\text{tube}}}{T_{\text{insp}\;\min}}\Rightarrow V_{\text{insp start}}=\frac{P_{\text{insp}\;\min}V_{\text{tube}}T_{\text{insp start}}}{T_{\text{insp}\;\min}P_{\text{insp start}}}$$ (2)

V_tube_ - V_insp start_ represents the maximum rebreathing volume originating from the expiratory tube.

At the start of expiration, all of the gas in the inspiratory tube is ideal gas, and the volume is *V_tube_*. Let the pressure and temperature of the gas be denoted as *P_exp start_* and *T_exp start_*, respectively (Figure 1E). When the pressure in the inspiratory tube reaches its maximum during the expiration, the pressure, volume, and temperature of the ideal gas are denoted as *P_exp max_*, *V_exp max_*, and *T_exp max_*, respectively (Figure 1F) (Equation 3).


$$\frac{P_{\text{expstart}}V_{\text{tube}}}{T_{\text{expstart}}}=\frac{P_{\text{expmax}}V_{\text{expmax}}}{T_{\text{expmax}}}\Rightarrow V_{\text{expmax}}=\frac{P_{\text{expstart}}V_{\text{tube}}T_{\text{expmax}}}{T_{\text{expstart}}P_{\text{expmax}}}$$ (3)

*V_tube_* - *V_exp max_* represents the maximum rebreathing volume originating from the inspiratory tube.

### 2.2 Ethics statement

The study included a 5-year-old male patient (18.6 kg, 109 cm) scheduled to undergo laparoscopic high ligation of an inguinal hernia sac under general anesthesia. Preoperative examinations showed no significant abnormalities, and the child had no respiratory infections or history of habitual snoring during sleep. This study was approved by the Ethics Committee of Children’s Hospital Affiliated to Chongqing Medical University (File No.: 2025–341). Informed consent was obtained from the child’s parents.

### 2.3 Ambient atmospheric pressure, critical pressure, and start pressure

Before the child entered the operating room, the temperature inside the operating room was set to 24 °C. After the ambient temperature stabilized, the ambient atmospheric pressure was measured with a barometer for 1 min under quiet conditions, and the average value was taken for calculating the absolute pressure.

**Figure 2:**
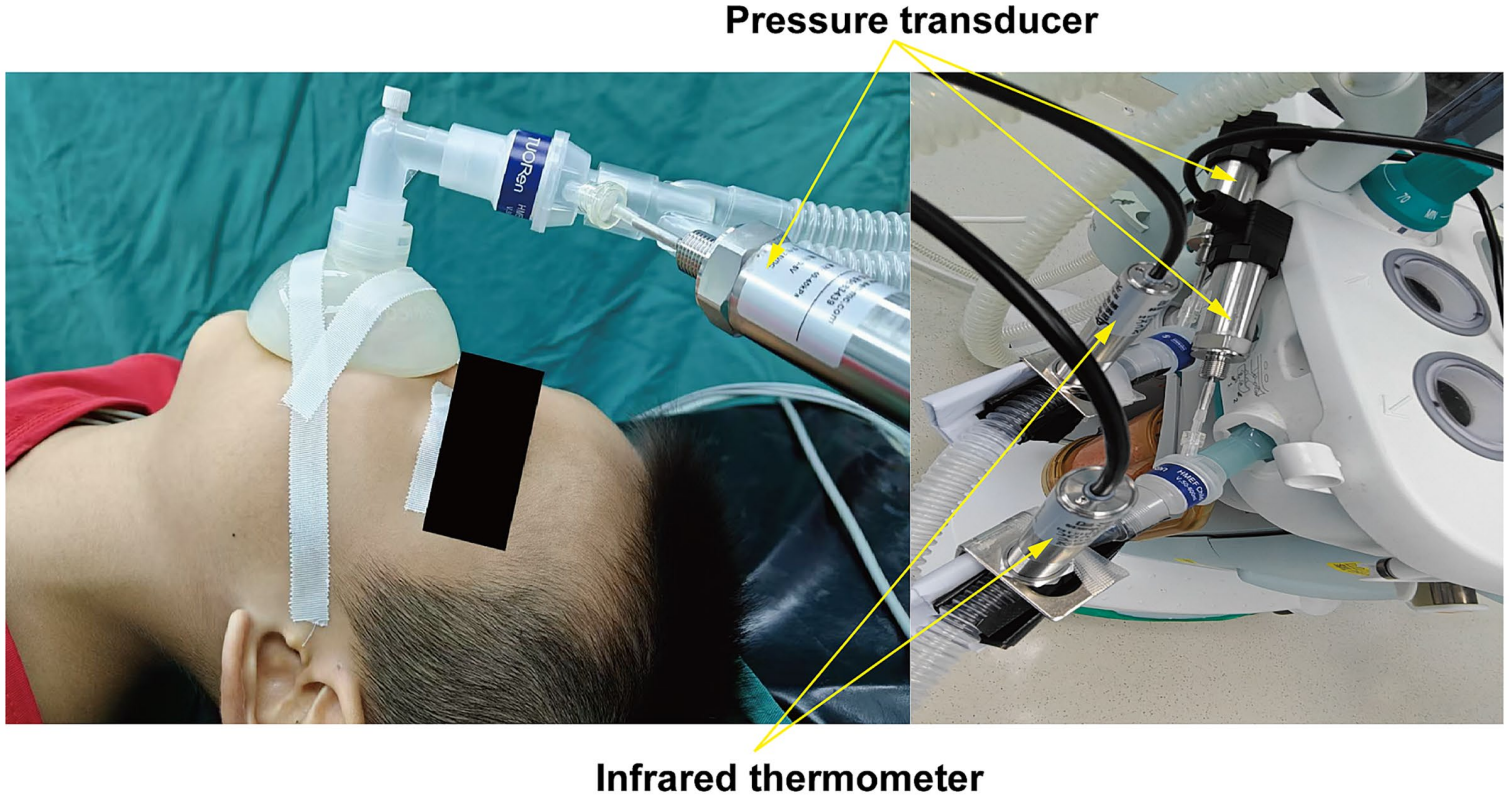
Measurement methods for gas pressure and temperature in the breathing tubing. (1) The pediatric breathing tubing we used is made of high-density polyethylene (HDPE) with a thermal conductivity of 0.35 ~ 0.45 W/(m·K) and a wall thickness of 0.7 ± 0.1 mm. Therefore, in theory, the tubing wall exhibits good thermal conduction performance. Two types of temperature probes with different operating principles could be used to measure the gas temperature inside the breathing tubing. The first is a probe that extends directly into the airflow and contacts the gas; the second is an infrared thermometer, which infers the internal gas temperature by measuring the wall temperature. In our preliminary experiments, we conducted a comparative study using these two types of probes simultaneously. Specifically, we placed both probes—with identical rated measurement ranges and accuracies—at similar positions on the breathing tubing. Under different flow rates, the actual temperature readings from the two types of probes differed by ≤0.1 °C. Based on these results, we ultimately selected the infrared thermometer because it does not interfere with the flowing gas in the main pathways. (2) In our preliminary experiments, we conducted a pre-test to determine the appropriate sampling frequency for pressure data by using two pressure transducers simultaneously—one set at 100 Hz and the other at 10 Hz. The two pressure transducers were placed close together on the expiratory/inspiratory tube. They were used to measure and compare the extreme values recorded at the key time points. We found that when the spontaneous breathing rate was below 50 breaths per minute, the difference between the values obtained at 100 Hz and those obtained at 10 Hz, divided by the 10 Hz value, was ≤ ± 1%. Therefore, we ultimately selected the pressure data acquisition scheme that generates less redundant data. Using a comparison protocol similar to that for the pressure transducers, we did not observe any meaningful difference in temperature readings between sampling frequencies of 100 Hz and 10 Hz. Consequently, we chose a sampling frequency of 10 Hz for temperature data as well.

Connected the Avance CS^2^ anesthesia workstation (Datex-Ohmeda, Madison, United States), the breathing tubing, the heat and moisture exchanger, and the mask. All pressure transducers (SCYG314, Wuxi, China, range: −4 to 4 kPa, full-scale accuracy: ±0.1%) were atmospherically zeroed before being attached to each of the three outlets of the breathing tubing. And the pressure transducers were placed on the wall of the breathing tubing (Figure 2). All gas pressure values mentioned in this study refer to static pressure. Connected an infrared thermometer (SA50A, Wuxi, China, range: 0 to 50 °C, accuracy: ±0.1 °C) to each of the two outlets of the breathing tubing near the anesthesia workstation (a black shield was placed on the opposite side of the infrared thermometer probe on the breathing tubing) (Figure 2). The mask was placed tightly against a smooth, flat surface with a fraction of inspired oxygen (FiO₂) of 30%. The pressure was measured at three points in the breathing tubing for 1 min with fresh gas flow of 2, 4, and 6 L/min, respectively, and their average values were calculated. The average pressure near the mask was used to determine the critical pressure (*P_critical_*) for the switching between inspiration and expiration during the child’s spontaneous breathing. The average pressure near the inspiratory port was used to determine the *P_exp start_* during the child’s spontaneous breathing. The average pressure near the expiratory port was used to determine the *P_insp start_* during the child’s spontaneous breathing.

Let the pressure near the mask be denoted as *P_mask_* during the child’s spontaneous breathing: when *P_mask_* fell below *P_critical_*, the child was in inspiration; when *P_mask_* rose above *P_critical_*, the child was in expiration. Theoretically, a complete inspiration process corresponded to *P_mask_* undergoing the full transition: *P_critical_* → minimum pressure → *P_critical_*. Similarly, a complete expiration process corresponded to *P_mask_* undergoing the full transition: *P_critical_* → maximum pressure → *P_critical_*.

### 2.4 Spontaneous breathing

After routine monitoring was established for the child, intravenous injections of midazolam (1.8 mg) and propofol (55 mg) were administered. Once the child fell asleep, spontaneous breathing was maintained via a mask at a fresh gas flow of 6 L/min with FiO₂ 30% until both respiratory rate and tidal volume nearly stabilized. While keeping FiO₂ at 30%, the child spontaneously breathed via the breathing tubing with fresh gas flow of 2, 4, and 6 L/min, each for 1 min, during which changes in gas states within the tubing and tidal volumes were synchronously recorded (Figure 2). Pressure and temperature changes in the breathing tubing were synchronously recorded using pressure transducers and infrared thermometers, respectively, at a data acquisition frequency of 10 Hz. Changes in tidal volume were synchronously recorded by video from the anesthesia workstation. Breathing counting and analysis began with the first complete inspiration observed and only performed analysis on complete breathing cycles.

### 2.5 Statistical analysis

Statistical analysis was performed using R software (version 4.1.1). Given that this study involved only a single subject, the three fresh gas flow conditions (2, 4, and 6 L/min) were introduced solely to test the method’s performance under different working conditions, rather than for intergroup comparison. All results are presented descriptively (mean ± standard deviation) by condition. The reporting of means and standard deviations is intended solely to illustrate the data dispersion and computational stability of the measurement-calculation method under varying fresh gas flows and consecutive repeated-breath conditions. No inferential hypothesis testing was performed, no *p*-values are reported, and the results carry no clinical extrapolation value.

## 3 Results

**Figure 3:**
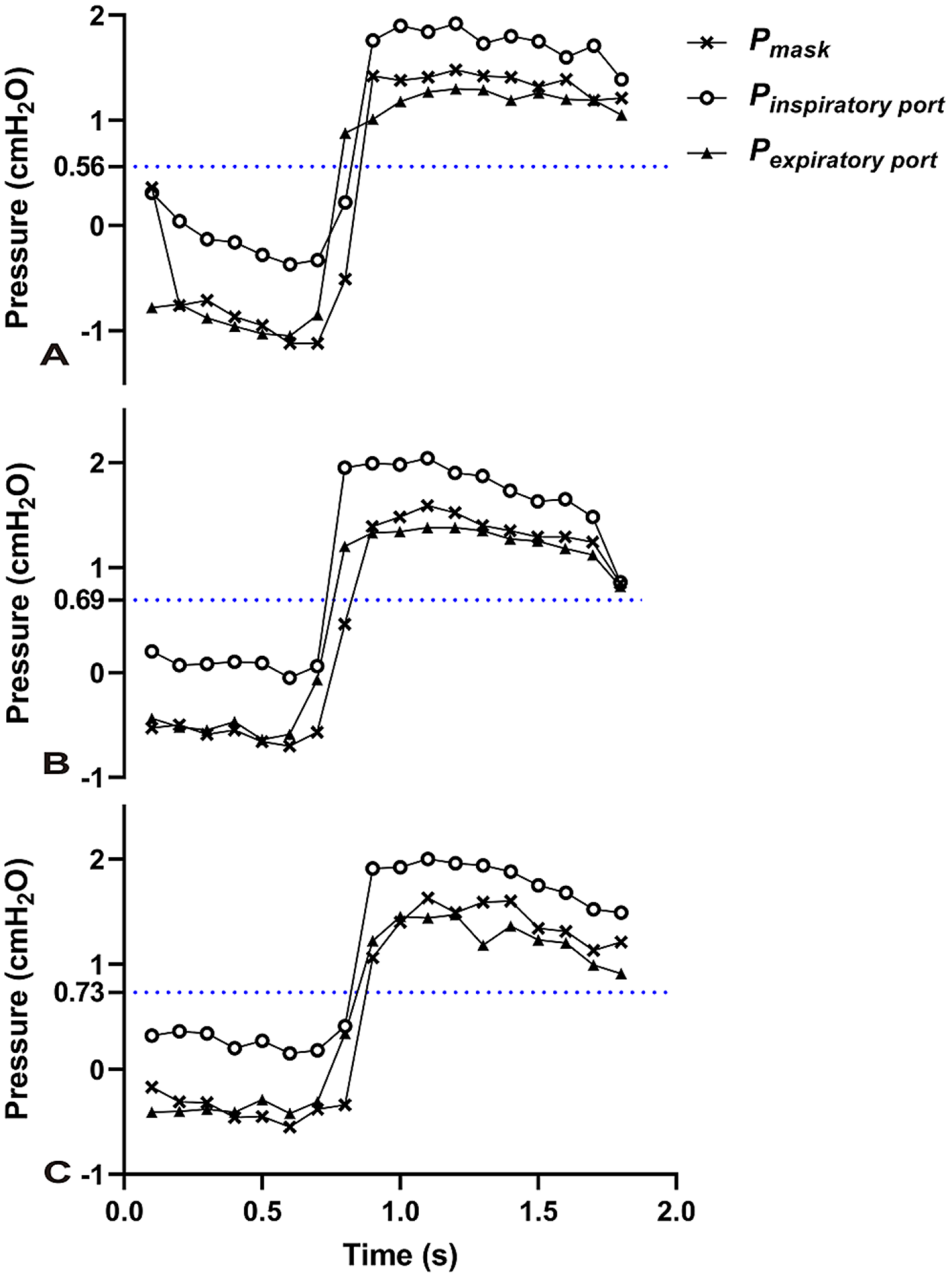
Pressure change of one breath in the breathing tubing during spontaneous breathing with different fresh gas flows. **(A)** 2 L/min; **(B)** 4 L/min; **(C)** 6 L/min. The blue dashed line represents the critical pressure corresponding to the fresh gas flow. *P_expiratory port_*, pressure near the expiratory port in the breathing tubing; *P_inspiratory port_*, pressure near the inspiratory port in the breathing tubing; *P_mask_*, pressure near the mask in the breathing tubing.

During the 1 min observation period, the number of captured effective breaths remained consistent at 33 across all tested fresh gas flows. The average duration of a single breath showed minimal numerical variation across the three fresh gas flow conditions (2, 4, and 6 L/min) (Table 1). Under varying fresh gas flow, similar pressure change trends occurred at identical locations within the breathing tubing during a single spontaneous breath cycle, and the transition between inspiration and expiration was rapid with nearly no pauses observed (Figure 3).

**Table 1:** Means ± standard deviation of breathing characteristics and the rebreathing volume during spontaneous breathing with fresh gas flow of 2, 4, and 6 L/min.

Fresh gas flow	2 L/min	4 L/min	6 L/min
Number of full breaths^a^	33	33	33
Time of a breath (s)	1.78 ± 0.06	1.75 ± 0.06	1.76 ± 0.06
Tidal volume (mL)	128.6 ± 13.5	111.2 ± 6.4	102.2 ± 3.7
Inspiratory-to-expiratory ratio	0.76 ± 0.06	0.82 ± 0.07	0.86 ± 0.09
Expiratory tube gas states (inspiration)			
*P_insp start_* (cmH_2_O)^b^	0.58	0.71	0.76
*T_insp start_* (°C) ^c^	25.01 ± 0.07	24.72 ± 0.05	24.08 ± 0.04
*P_insp min_* (cmH_2_O)^b^	−0.99 ± 0.15	−0.63 ± 0.09	−0.45 ± 0.08
*T_insp min_* (°C)^c^	24.91 ± 0.06	24.63 ± 0.05	23.98 ± 0.05
*T_insp start_* - *T_insp min_* (°C)	0.09 ± 0.02	0.09 ± 0.02	0.09 ± 0.02
*V_tube_* - *V_insp start_* (mL)	0.29 ± 0.04	0.23 ± 0.03	0.20 ± 0.02
Inspiratory tube gas states (expiration)			
*P_exp start_* (cmH_2_O)^b^	0.81	0.95	1.01
*T_exp start_* (°C) ^c^	23.80 ± 0.02	23.60 ± 0.02	23.40 ± 0.02
*P_exp max_* (cmH_2_O)^b^	1.91 ± 0.08	1.97 ± 0.08	2.02 ± 0.06
*T_exp max_* (°C) ^c^	23.80 ± 0.02	23.60 ± 0.02	23.40 ± 0.02
*T_exp start_* - *T_exp max_* (°C)	0	0	0
*V_tube_* - *V_exp max_* (mL)	0.25 ± 0.02	0.23 ± 0.02	0.23 ± 0.01
Rebreathing from the breathing tubing			
(*V_tube_* - *V_insp start_*) + (*V_tube_* - *V_exp max_*) (mL)	0.54 ± 0.05	0.47 ± 0.03	0.43 ± 0.03
Rebreathing fraction (%)^d^	0.42 ± 0.03	0.42 ± 0.03	0.42 ± 0.03

a Breathing counting and analysis began with the first complete inspiration observed and only performed analysis on complete breathing cycles.

b Measured pressure values must be converted to absolute pressure in the calculation process by adding the actual ambient atmospheric pressure measured in the operating room (97.57 kPa).

c Measured temperature values must be converted to absolute temperature in the calculation process by adding 273.15 °C.

d Rebreathing fraction equals the maximum rebreathing volume originating from the breathing tubing divided by tidal volume.

*P_exp max_*, the maximum pressure in the inspiratory tube during expiration; *P_exp start_*, the pressure in the inspiratory tube at the start of expiration; *P_insp min_*, the minimum pressure in the expiratory tube during inspiration; *P_insp start_*, the pressure in the expiratory tube at the start of inspiration; *T_exp max_*, the temperature of the inspiratory tube at maximum pressure during expiration; *T_exp start_*, the temperature of the inspiratory tube at the start of expiration; *T_insp min_*, the temperature of the expiratory tube at minimum pressure during inspiration; *T_insp start_*, the temperature of the expiratory tube at the start of inspiration; *V_exp max_*, the volume of ideal gas in the inspiratory tube at maximum pressure during expiration; *V_insp start_*, the volume of ideal gas in the expiratory tube at the start of inspiration; *V_tube_*, the volume of the inspiratory/expiratory tube; *V_tube_* - *V_exp max_*, the maximum rebreathing volume originating from the inspiratory tube; *V_tube_* - *V_insp start_*, the maximum rebreathing volume originating from the expiratory tube.

### 3.1 Inspiration in expiratory tube

**Figure 4:**
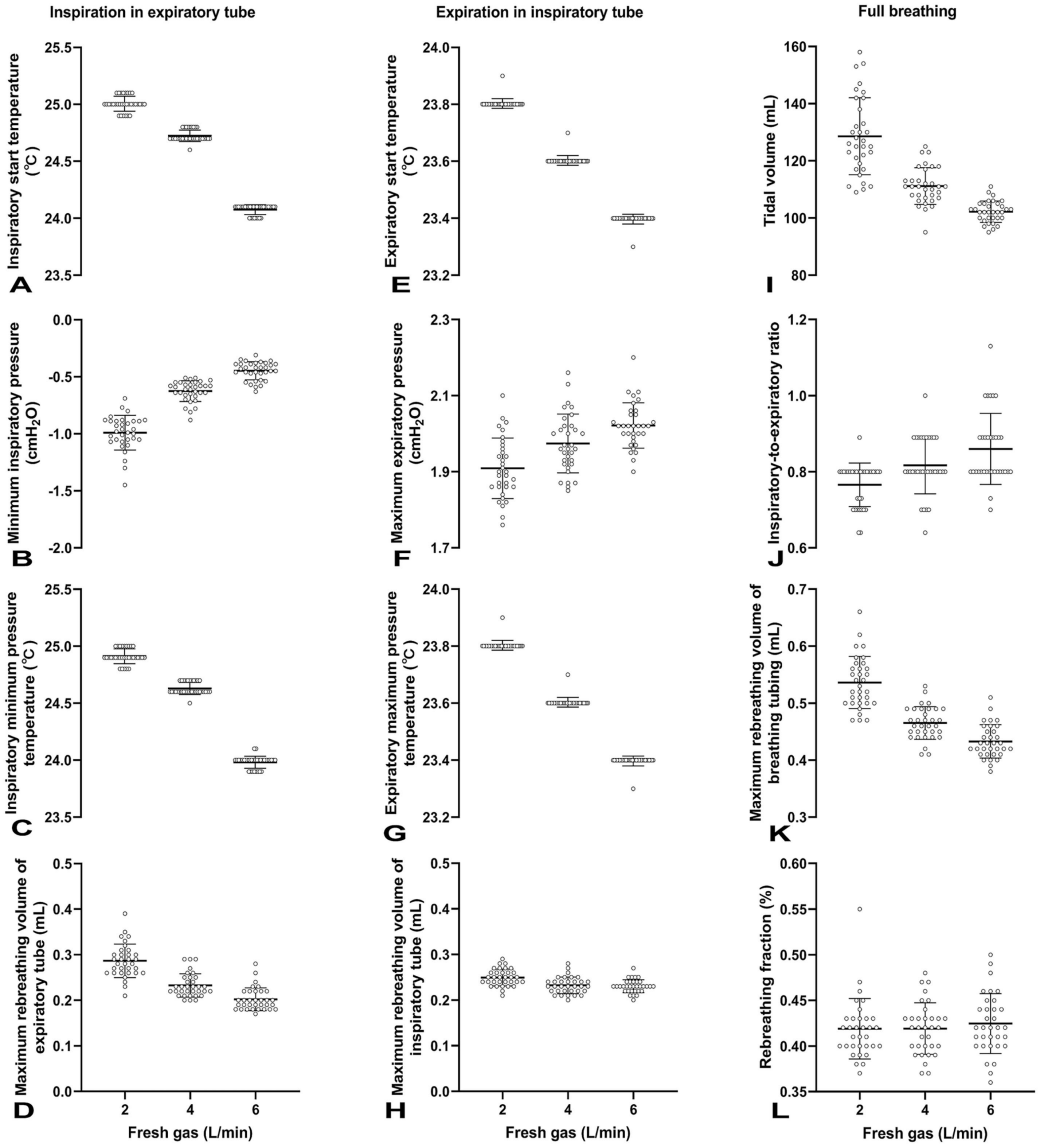
Parameters plotted against fresh gas flow during spontaneous breathing via the circle breathing system. **(A)** Inspiratory start temperature; **(B)** Minimum inspiratory pressure; **(C)** Inspiratory minimum pressure temperature; **(D)** Maximum rebreathing volume of expiratory tube; **(E)** Expiratory start temperature; **(F)** Maximum expiratory pressure; **(G)** Expiratory maximum pressure temperature; **(H)** Maximum rebreathing volume of inspiratory tube; **(I)** Tidal volume; **(J)** Inspiratory-to-expiratory ratio; **(K)** Maximum rebreathing volume of breathing tubing; **(L)** Rebreathing fraction. Error bars represent the standard deviation.

At fresh gas flows of 2, 4, and 6 L/min, the start temperatures during inspiration were 25.01 ± 0.07 °C, 24.72 ± 0.05 °C, and 24.08 ± 0.04 °C, respectively, showing a decreasing trend with increasing fresh gas flow (Table 1; Figure 4A). The minimum pressures during inspiration were −0.99 ± 0.15 cmH_2_O, −0.63 ± 0.09 cmH_2_O, and −0.45 ± 0.08 cmH_2_O, respectively, showing an increasing trend with increasing fresh gas flow (Table 1; Figure 4B). The temperatures at the minimum pressure during inspiration were 24.91 ± 0.06 °C, 24.63 ± 0.05 °C, and 23.98 ± 0.05 °C, respectively, also decreasing with increasing fresh gas flow (Table 1; Figure 4C). Within each fresh gas flow condition, the mean difference between the temperature at minimum pressure and the start temperature was identical, with a value of 0.09 ± 0.02 °C. The maximum rebreathing volumes originating from the expiratory tube were 0.29 ± 0.04 mL, 0.23 ± 0.03 mL, and 0.20 ± 0.02 mL, respectively, showing a decreasing trend with increasing fresh gas flow (Table 1; Figure 4D).

### 3.2 Expiration in inspiratory tube

At fresh gas flows of 2, 4, and 6 L/min, the start temperatures during expiration were 23.80 ± 0.02 °C, 23.60 ± 0.02 °C, and 23.40 ± 0.02 °C, respectively, showing a decreasing trend with increasing fresh gas flow (Table 1; Figure 4E). The maximum pressures during expiration were 1.91 ± 0.08 cmH_2_O, 1.97 ± 0.08 cmH_2_O, and 2.02 ± 0.06 cmH_2_O, respectively, showing an increasing trend with increasing fresh gas flow (Table 1; Figure 4F). The temperatures at maximum pressure during expiration were 23.80 ± 0.02 °C, 23.60 ± 0.02 °C, and 23.40 ± 0.02 °C, respectively, also decreasing with increasing fresh gas flow (Table 1; Figure 4G). Within each fresh gas flow condition, the mean difference between the temperature at maximum pressure and the start temperature during expiration was consistently zero. The maximum rebreathing volumes originating from the inspiratory tube were 0.25 ± 0.02 mL, 0.23 ± 0.02 mL, and 0.23 ± 0.01 mL, respectively, showing a decreasing trend with increasing fresh gas flow (Table 1; Figure 4H).

### 3.3 Full breathing

At fresh gas flows of 2, 4, and 6 L/min, the tidal volumes were 128.6 ± 13.5 mL, 111.2 ± 6.4 mL, and 102.2 ± 3.7 mL, respectively, showing a decreasing trend with increasing fresh gas flow (Table 1; Figure 4I). The inspiratory-to-expiratory ratios were 0.76 ± 0.06, 0.82 ± 0.07, and 0.86 ± 0.09, respectively, showing an increasing trend with increasing fresh gas flow (Table 1; Figure 4J). The total maximum rebreathing volumes originating from the breathing tubing were 0.54 ± 0.05 mL, 0.47 ± 0.03 mL, and 0.43 ± 0.03 mL, respectively, showing a decreasing trend with increasing fresh gas flow (Table 1; Figure 4K). The rebreathing fraction—defined as the ratio of the sum of rebreathing from the expiratory tube and inspiratory tube to tidal volume—was 0.42 ± 0.03%, 0.42 ± 0.03%, and 0.42 ± 0.03%, respectively, showing no obvious trend with increasing fresh gas flow (Table 1; Figure 4L).

**Figure 5:**
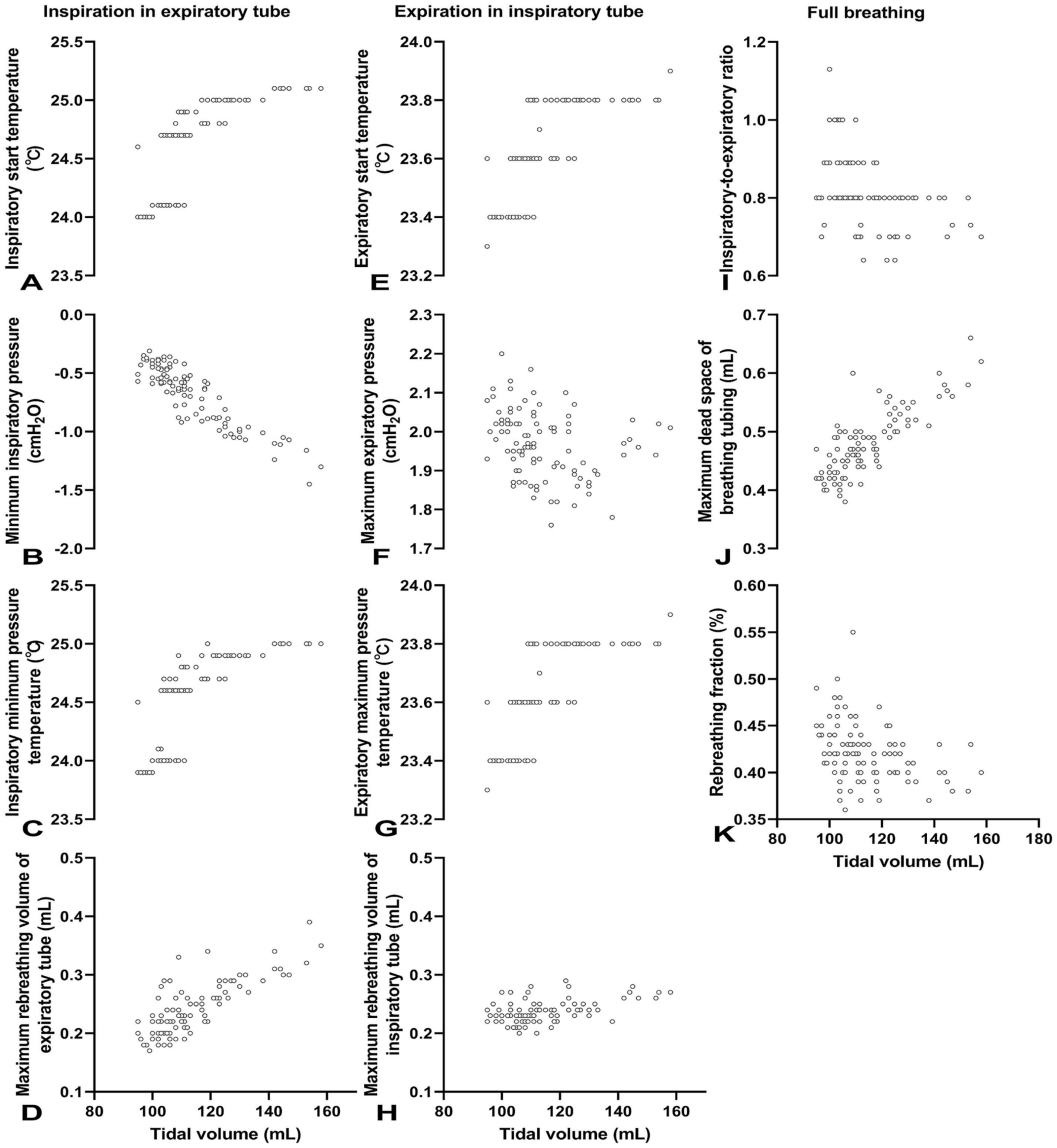
Parameters plotted against tidal volume during spontaneous breathing via the circle breathing system. **(A)** Inspiratory start temperature; **(B)** Minimum inspiratory pressure; **(C)** Inspiratory minimum pressure temperature; **(D)** Maximum rebreathing volume of expiratory tube; **(E)** Expiratory start temperature; **(F)** Maximum expiratory pressure; **(G)** Expiratory maximum pressure temperature; **(H)** Maximum rebreathing volume of inspiratory tube; **(I)** Inspiratory-to-expiratory ratio; **(J)** Maximum dead space of breathing tubing; **(K)** Rebreathing fraction.

The changing trend of the states of the gas in the expiratory tube with tidal volume during inspiration is similar to that of the states of the gas in the inspiratory tube with tidal volume during expiration (Figures 5A–H). The inspiratory-to-expiratory ratio showed a decreasing trend with increasing tidal volume (Figure 5I). Additionally, the total maximum rebreathing volumes originating from the breathing tubing showed an increasing trend with increasing tidal volume (Figure 5J), while the rebreathing fractions showed a decreasing trend with increasing tidal volume (Figure 5K).

## 4 Discussion

This proof-of-concept study proposed and demonstrated the feasibility of a novel method that is minimally intrusive with the gas flow to quantify the rebreathing originating from the breathing tubing during spontaneous breathing via the circle breathing system. This method relied on real-time monitoring of gas pressure and temperature on the wall of the breathing tubing, coupled with specific formulas derived from the ideal gas law. Our preliminary data from a single subject demonstrate that this approach can sensitively detect flow-dependent changes in calculated rebreathing volume, establishing its fundamental feasibility.

It is important to emphasize that all numerical trends reported in this study were derived from repeated measurements within a single subject. As such, they reflect the operational characteristics of the measurement method under specific conditions rather than any population-level physiological rules. Thus, these results should be interpreted strictly within the context of feasibility testing.

The primary contribution of this work is methodological. While prior studies have successfully modeled system-wide rebreathing using steady-state assumptions (8, 19), the specific behavior of the small bidirectional component originating in the breathing tube has not previously been amenable to direct measurement (20, 21). Our method resolves this component and reveals that its dynamic behavior is more characteristic of a cyclic minor overflow than a constant stream. This observation does not contradict existing models; rather, it describes a compartment that those models were never designed to characterize. This study provides the first quantitative description of that compartment and the measurement framework required for its future investigation.

Previous research had shown that with an increase in fresh gas flow, breathing characteristics, such as tidal volume, were altered due to the elevated pressure in the circle breathing system (22). In this study, the child’s spontaneous breathing characteristics exhibited regular variations as the fresh gas flow increased, and such regular variations could be captured by the measurement-calculation method investigated in this research. Previous studies have demonstrated that elevated pressure within the circle breathing system may impede the expiratory process in patients, requiring the patients to exert greater expiratory effort and potentially shortening the expiratory time (23). Our study yielded consistent changing trends. Whether such changes resulted in more gas remaining in the lungs at the end of expiration, the impact of this phenomenon on alveolar ventilation requires further investigation.

The real-time measurements of gas pressure and temperature within the breathing tubing showed distinct and regular variations with changes in fresh gas flow. And the calculated total maximum rebreathing volume from the breathing tubing showed a decreasing trend with increasing fresh gas flow. This finding was consistent with prior research demonstrating that elevated fresh gas flow enhanced the elimination of carbon dioxide from the system (24). However, it was noteworthy that the absolute value of the maximum rebreathing volume originating from the breathing tubing calculated by the formulas was relatively small, far less than the rebreathing introduced by the heat and moisture exchanger commonly used in the circle breathing system (25). Moreover, its ratio to tidal volume (rebreathing fraction) remained well below the critical thresholds of concern reported in previous reports (26, 27). Since the data in this study were obtained from a single subject, the findings cannot truly and accurately reflect population-level data beyond the validation of the measurement-calculation method itself. Further investigations are therefore required to clarify the clinical implications of these results.

In summary, our study provided a feasible, direct monitoring, and highly sensitive method for quantifying the rebreathing originating from the breathing tubing by leveraging the physical states of the gas during spontaneous breathing. The primary advantages are as follows: First, the wall-mounted sensors minimize interference with gas flow. Second, the setup is compatible with existing disposable tubing. Third, the method offers a direct and sensitive quantification of the rebreathing originating from the breathing tubing during spontaneous breathing, which has been notoriously difficult to isolate and measure.

This study has some limitations, which should be acknowledged. Single-subject designs can only be used to clarify the operational feasibility of testing methods; generalized conclusions require further research. The absence of a recognized gold standard for comparison validation continues to be a limitation. Additionally, the critical pressure was measured before the child was connected to the breathing tubing. Although theoretically the critical pressure value was expected to fall within a complete inspiration or expiration, its precise value may not be accurately recorded due to the sampling interval. This study establishes a measurement framework for quantifying rebreathing that originates from the breathing tubing, and the method appears feasible in this preliminary setting. Determining whether this variable contributes to physiological outcomes is beyond the scope of this feasibility report and will require future studies that involve diverse ages and respiratory mechanics profiles using this methodology.

## 5 Conclusion

In conclusion, we have developed and provided preliminary evidence for a new method to quantify the rebreathing originating from the breathing tubing in the circle breathing system based on the ideal gas law during spontaneous breathing. It offers a tool to objectively assess a previous lack of concrete understanding in rebreathing by monitoring real-time gas states. Future research should focus on validating the stability of this method using a mechanical lung model and further verify it across a broader population (with varying ages and body weights) to define its full clinical utility and potential role in optimizing ventilation strategies during spontaneous breathing anesthesia.

## Data Availability

The raw data supporting the conclusions of this article will be made available by the authors, without undue reservation.
